# Insulin Sensitivity Is Associated with Lipoprotein Lipase (*LPL*) and Catenin Delta 2 (*CTNND2*) DNA Methylation in Peripheral White Blood Cells in Non-Diabetic Young Women

**DOI:** 10.3390/ijms20122928

**Published:** 2019-06-15

**Authors:** Ana Arpón, José L. Santos, Fermín I. Milagro, Luis Rodrigo Cataldo, Carolina Bravo, José-Ignacio Riezu-Boj, J. Alfredo Martínez

**Affiliations:** 1 Department of Nutrition, Food Sciences and Physiology and Centre for Nutrition Research, University of Navarra, 31008 Pamplona, Spain; aarpon.1@alumni.unav.es (A.A.); fmilagro@unav.es (F.I.M.); jiriezu@unav.es (J.-I.R.-B.); jalfmtz@unav.es (J.A.M.); 2 Department of Nutrition, Diabetes and Metabolism, School of Medicine, Pontificia Universidad Católica de Chile, Santiago 8331150, Chile; rodrigo.cataldo_buscunan@med.lu.se (L.R.C.); carobravo.vasquez@gmail.com (C.B.); 3 Navarra Institute for Health Research (IdiSNa), 31008 Pamplona, Spain; 4 Centro de Investigación Biomédica en Red Fisiopatología de la Obesidad y Nutrición (CIBERobn), Instituto de Salud Carlos III, 28029 Madrid, Spain; 5 Precision Nutrition and Cardiometabolic Health Program, Madrid Institute for Advanced Studies (IMDEA), IMDEA Food, 28049 Madrid, Spain

**Keywords:** insulin resistance, diabetes, epigenetics, insulin sensitivity index, EWAS

## Abstract

Hyperglycaemia and type 2 diabetes (T2D) are associated with impaired insulin secretion and/or insulin action. Since few studies have addressed the relation between DNA methylation patterns with elaborated surrogates of insulin secretion/sensitivity based on the intravenous glucose tolerance test (IVGTT), the aim of this study was to evaluate the association between DNA methylation and an insulin sensitivity index based on IVGTT (calculated insulin sensitivity index (CSi)) in peripheral white blood cells from 57 non-diabetic female volunteers. The CSi and acute insulin response (AIR) indexes, as well as the disposition index (DI = CSi × AIR), were estimated from abbreviated IVGTT in 49 apparently healthy Chilean women. Methylation levels were assessed using the Illumina Infinium Human Methylation 450k BeadChip. After a statistical probe filtering, the two top CpGs whose methylation was associated with CSi were cg04615668 and cg07263235, located in the catenin delta 2 (*CTNND2*) and lipoprotein lipase (*LPL*) genes, respectively. Both CpGs conjointly predicted insulin sensitivity status with an area under the curve of 0.90. Additionally, cg04615668 correlated with homeostasis model assessment insulin-sensitivity (HOMA-S) and AIR, whereas cg07263235 was associated with plasma creatinine and DI. These results add further insights into the epigenetic regulation of insulin sensitivity and associated complications, pointing the *CTNND2* and *LPL* genes as potential underlying epigenetic biomarkers for future risk of insulin-related diseases.

## 1. Introduction

Diabetes is defined as “a group of metabolic diseases characterized by hyperglycaemia resulting from defects in insulin secretion, insulin action, or both” [1]. Although the relative contribution of insulin secretion versus insulin action impairments in type 2 diabetes (T2D) depends on many factors, it has been extensively reported that obesity-related insulin resistance plays an important role in the onset and development of T2D [2]. Insulin resistance (or its inverse, insulin sensitivity) shows high inter-individual variability. Therefore, it is important to assess the performance of biomarkers of insulin sensitivity in the absence of hyperglycaemia, before inflammation and other obesity-related impairments of metabolism appear, in order to adequately evaluate the initial stages and directionality of the relation between the proposed biomarkers with insulin sensitivity. Epidemiologic studies have focused on simple measurements of insulin sensitivity based on glucose and insulin fasting plasma samples, such as the homeostasis model assessment insulin-sensitivity (HOMA-S) index, which is the inverse of the commonly used HOMA-insulin resistance (HOMA-IR) index [3]. Given that the main contributor of circulating glucose in fasting conditions is the liver, it is generally accepted that the HOMA-S index predominantly represents a measure of hepatic insulin sensitivity [4]. In contrast, other insulin sensitivity measurements, such as the M-value of the hyperinsulinemic-euglycaemic clamp, are obtained under conditions where a constant high level of circulating insulin is maintained and then, endogenous hepatic glucose production is inhibited [5]. Thus, the M-value can be considered mainly a measure of systemic and/or muscle/adipose insulin sensitivity. Alternatively, other general measures of insulin sensitivity have been derived from the oral glucose tolerance test (OGTT), that allows the calculation of the Matsuda-ISICOMP index and other insulin-related indexes, or from the intravenous glucose tolerance test (IVGTT) [6]. The IVGTT is a procedure that has the interesting operational advantage of allowing the simultaneous measurement of insulin secretion and insulin sensitivity in the same test [7,8]. The specific use of an abbreviated version of the IVGTT (1 hour test, instead of the extended 3 hour IVGTT test) provides gold-standard measurements of acute insulin release (AIR), using the area under the curve (AUC) of plasma insulin during the first 10 min of the IVGTT, and adequate estimations of insulin sensitivity through the calculated insulin sensitivity (CSi), using the plasma insulin and glucose measurements during the second part, 10 to 50 min of the abbreviated IVGTT [9]. Interestingly, a hyperbolic relation has been described for insulin secretion and sensitivity indexes derived from IVGTT in such a way that it is possible to calculate the disposition index (DI) as the product between insulin secretion and insulin sensitivity (DI = AIR × CSi) [7,10]. DI is considered a measure of insulin secretion adjusted by systemic insulin sensitivity representing a marker of glucose homeostasis dysregulation [11]. Additionally, it has been reported that both DI and the oral disposition index (ODI) based on OGTT are relevant predictors of future T2D development [12].

In the prediabetes status, the increased plasma glucose levels and the hyperinsulinemia are triggered by a failure in normal glucose homeostasis that have been related, among many other factors, with transcriptional variations in key metabolic organs that may be explained by epigenetic regulation [13]. Indeed, epigenome-wide association studies (EWASs) have revealed an influence of DNA methylation in genes related to T2D and glucose homeostasis [14,15,16,17,18,19,20]. These changes directly influence both insulin-producing pancreatic β-cells, as well as other organs involved in glucose homeostasis. Changes in methylation patterns related to T2D development are also accompanied by variations of methylation patterns in blood cells [21,22]. There are no studies in the literature analysing the relations between leukocyte DNA methylation across the genome and insulin sensitivity measured by IVGTT or studies specifically focused on the DI.

Since diabetes is not usually diagnosed until several years after the appearance of insulin and glucose deregulation, it is crucial to detect the early stages of the disease through the use of adequate biomarkers of reduced insulin sensitivity [23]. In this context, studies conducted in non-diabetic subjects are useful in evaluating novel biomarkers to identify the susceptibility to develop T2D through the evaluation of intermediate phenotypes such as the insulin sensitivity. Therefore, the aim of this study was to assess the association between DNA methylation patterns in peripheral white blood cells (PWBCs) with measures of insulin sensitivity based on the intravenous glucose tolerance tests in non-diabetic women.

## 2. Results

### 2.1. Anthropometric and Biochemical Characteristics of the Participants

Summary statistics for anthropometric and biochemical variables, as well as insulin sensitivity measurements, are reported in Table 1.

### 2.2. CpG Sites Selection and Ingenuity Pathway Analysis

**Figure 1:**
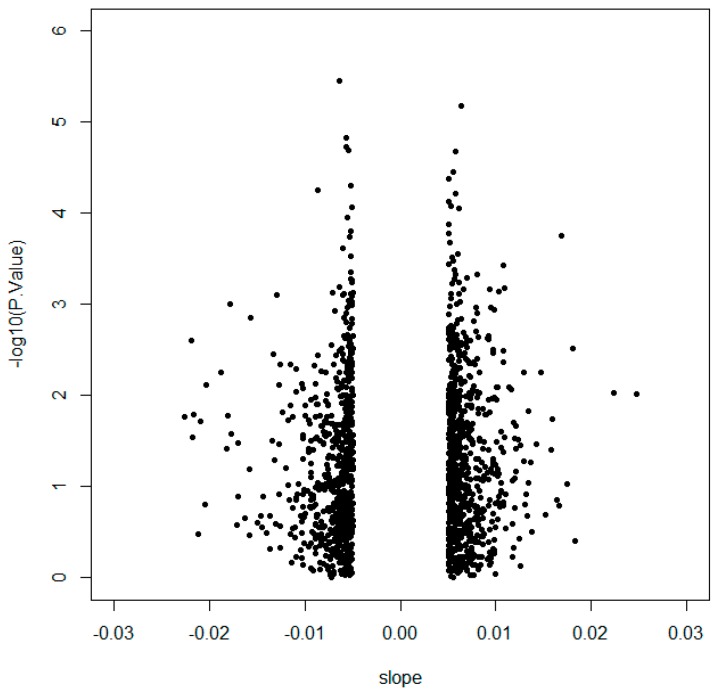
Volcano plot representing 1416 CpGs selected by slope >|0.005| according to the logarithm of the *p*-value obtained from Spearman correlation with calculated insulin sensitivity index (CSi).

**Figure 2:**
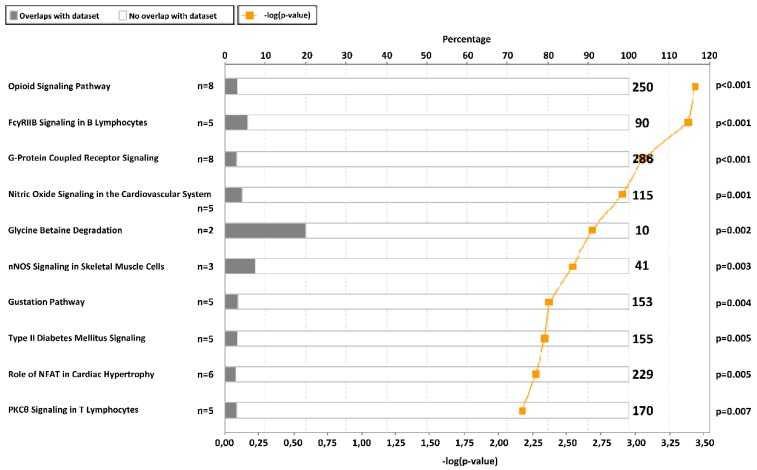
Top 10 canonical pathways from ingenuity pathway analysis of 253 CpGs selected by Spearman false discovery rate (FDR) < 0.05. The graph presents the canonical pathways ordered by −log(*p*-value) and the percentage of genes from our list that are in one specific pathway (total number of genes in the pathway at the right part of the graph). The *p*-value adjusted by Fisher’s test is also indicated at the right side.

In order to identify the CpG sites with the highest methylation variability that may have a biological implication, an initial selection by the slope between methylation and CSi was performed to discard multiple CpG sites showing lack of intrinsic variation. Then, 1416 CpGs with a slope >|0.005| were further analysed (Figure 1, Table S1) because of their correlation with CSi. The raw *p*-values from non-parametric correlational analysis were subsequently adjusted by the Benjamini–Hochberg method, resulting in 253 CpG sites significantly associated with CSi (false discovery rate (FDR) < 0.05) (Table S2). These 253 CpGs were analysed for canonical pathways from Ingenuity Pathway Analysis (IPA) (Table S3). Some of the obtained canonical pathways were related to insulin and glucose (Figure 2), such as opioid signalling pathway, G-protein coupled receptor, glycine betaine degradation, nitric oxide signalling in the cardiovascular system, gustation pathway or type 2 diabetes mellitus.

**Figure 3:**
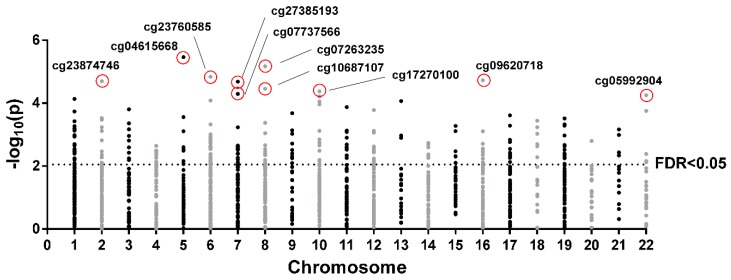
Manhattan plot of 1416 CpGs selected by slope >|0.005| in each chromosome. Points above the horizontal line are false discovery rate (FDR) < 0.05. The top 10 CpGs are indicated.

**Figure 4:**
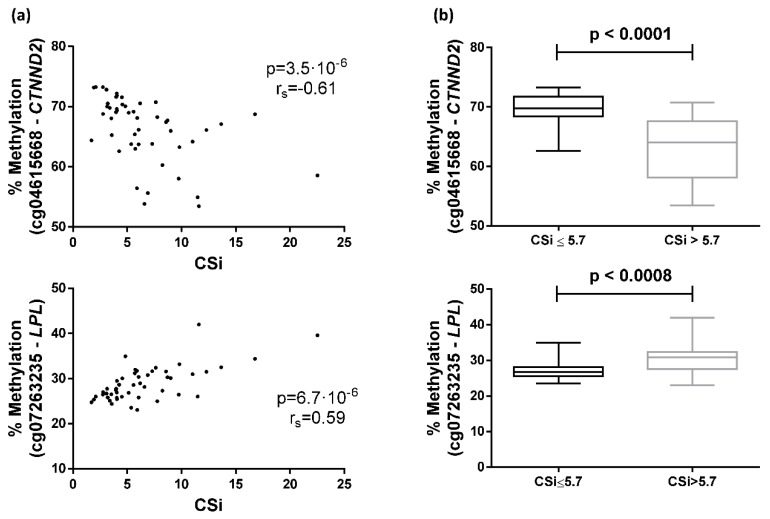
Associations between the calculated insulin sensitivity index (CSi) and DNA methylation in peripheral white blood cells. (**a**) Spearman correlation between CSi and cg04615668-*CTNND2* or cg07263235-*LPL* methylation; (**b**) Differential methylation of cg04615668-*CTNND2* or cg07263235-*LPL* between individuals separated by the median of CSi.

The 10 top most significant CpGs of the 253 CpGs selected by FDR < 0.05 were cg04615668-*CTNND2* (corresponding gene according to the Illumina CG Database), cg07263235-*LPL*, cg09620718-*ACSM1*, cg23760585-*FLJ22536*, cg23874746-*PDE1A*, cg27385193-NA, cg10687107-NA, cg17270100-NA, cg07737566-*GRB10*, and cg05992904-*FAM19A5* (Figure 3, Figure S1). Further analyses where performed with the two most significant CpGs, cg04615668 and cg07263235, which are located in the genes catenin delta 2 (*CTNND2*) and lipoprotein lipase (*LPL*), respectively. Correlations between DNA methylation and CSi for both CpGs are plotted (Figure 4A).

### 2.3. Differences between Groups Separated by the Median of CSi Values

Participants of the GEDYMET (genetics, dysglycemia and metabolism) study were also separated by the median CSi values (cut-off value = 5.7) to categorise the subjects into insulin-sensitive (higher CSi values, *n* = 24) and insulin-resistant (lower CSi values, *n* = 25) groups. The group with higher CSi showed a methylation mean and SD of 63.2(5.4) for cg04615668 and 30.7(4.2) for cg07263235, whereas the group with lower CSi presented a methylation mean and SD of 69.4(3.0) and 27.1(2.4) for cg04615668 and cg07263235, respectively. Significant differences in methylation percentage of cg04615668 and cg07263235 were found when comparing both groups (Figure 4B).

**Figure 5:**
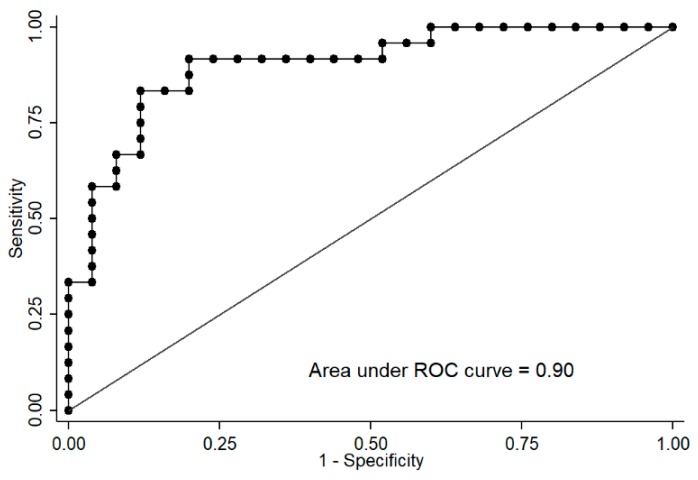
Receiver operating characteristic (ROC) curve of the logistic regression of cg04615668-*CTNND2* and cg07263235-*LPL* adjusted by sex allows the discrimination of subjects with the calculated insulin sensitivity index (CSi) ≤5.7 (insulin-resistant) versus >5.7 (insulin-sensitive).

Additionally, logistic regressions and receiver operating characteristic (ROC) curves, both adjusted by age, were carried out to determine whether both CpG site methylation levels were able to predict the CSi group. Logistic regressions showed an odds ratio (OR) = 0.67 for cg04615668 (pseudo *R*^2^ = 0.34, *p* < 0.0001) and OR = 1.43 for cg07263235 (pseudo *R*^2^ = 0.21, *p* = 0.0009). The AUCs were estimated as 0.86 (95% confidence interval 0.75–0.96) for cg04615668 and 0.81 (95% confidence interval 0.68–0.94) for cg07263235. Interestingly, multiple logistic regression including both CpGs adjusted by age significantly improved the model (OR cg04615668 = 0.68, OR cg07263235 = 1.36, pseudo *R*^2^ = 0.44, *p* < 0.0001), reaching an AUC of 0.90 for predicting CSi (Figure 5).

### 2.4. Correlation with Other Variables

**Figure 6:**
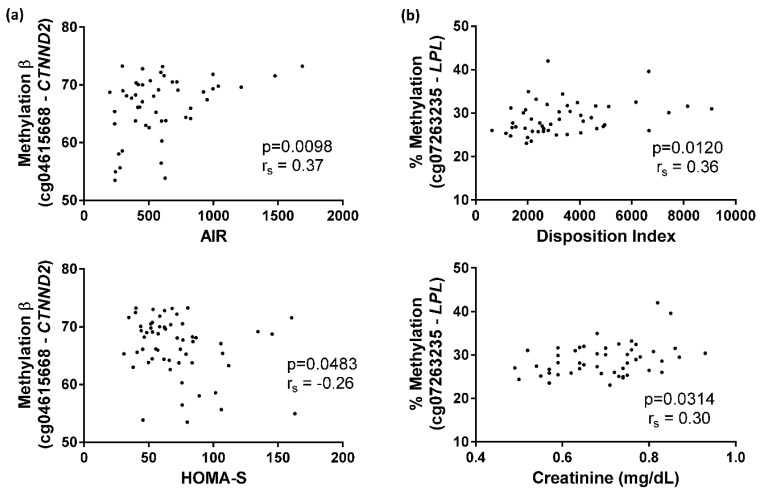
Spearman correlations of cg04615668 and cg07263235 methylation. (**a**) Correlation between cg04615668-*CTNND2* methylation and acute insulin response (AIR) index or homeostasis model assessment insulin-sensitivity (HOMA-S) index; (**b**) Correlation between cg07263235-*LPL* methylation and disposition index (DI) or plasma creatinine.

Furthermore, methylation values at the cg04615668 site significantly correlated with AIR (*p* = 0.0098) and HOMA-S (*p* = 0.0483) (Figure 6A), while methylation values at the cg07263235 site were significantly associated with plasma creatinine (*p* = 0.0314) and DI (*p* = 0.0120) (Figure 6B).

## 3. Discussion

The CpG sites cg04615668 and cg07263235, located in the *CTNND2* and *LPL* genes, respectively, achieved the most significant signals of association between DNA methylation levels in PWBCs and IVGTT-based insulin sensitivity measurements (CSi). Methylation of these specific CpGs was clearly different in the two groups separated by the median CSi of the whole group. Furthermore, both CpGs together were able to predict CSi using ROC curve analysis. The CpG cg04615668 was also associated with the AIR and HOMA-S indexes, whereas cg07263235 was correlated with the DI (defined as the product between AIR × CSi) and plasma creatinine levels. To our knowledge, this study is the first to relate CSi with DNA methylation, adding further insights into the epigenetic regulation of systemic insulin sensitivity and related traits. 

There is a need to develop biomarkers to detect early steps in the pathophysiologic progression of T2D, as well as to elucidate underlying mechanisms of the disease [24]. Genetics, epigenetics, as well as non-genetic factors (diet, lifestyle) are involved in the pathogenesis of dysglycaemia and T2D [25]. On the other hand, deregulations in insulin sensitivity and secretion might be associated with epigenetic modifications [13]. Previous EWAS showed an association between DNA methylation patterns in PWBCs and T2D and glucose homeostasis traits [14,15,16,17,18,19,20]. Additionally, different studies have proposed potential DNA methylation biomarkers in relation to plasma insulin levels, insulin secretion and insulin resistance such as those located in *PPARGC1A*, *HTR2A*, *LY86*, *TFAM*, *GIPR*, *ADIPOQ*, and *IGFBP3* genes [21]. Our study has found a relation between the insulin sensitivity index CSi, based on IVGTT [9], and methylation of CpGs in several genes. According to IPA, some of these genes were related to insulin-related pathways and T2D signalling, such as type 2 diabetes mellitus signalling. In the case of the opiod signalling pathway, opioid µ-receptors may be activated by β-endorphin to improve insulin resistance [26] and opiates can inhibit insulin signalling through direct crosstalk between the downstream signalling pathways of the opioid receptor and the insulin receptor [27]. As for the G-protein coupled receptor signalling, insulin and glucagon secretion is affected by factors binding to G-protein coupled receptors on the surface of β- and α-cells [28]. Regarding the glycine betaine degradation pathway, glycine betaine improves glucose tolerance and has been associated with reduced incidence of diabetes [29]. Furthermore, the pathway nitric oxide signalling in the cardiovascular system involves nitric oxide, which represents a central regulator of energy metabolism and body composition [30], and it is also a component of the insulin-signalling cascade [31]. The gustation pathway may also be related since inhibition of sweet chemosensory receptors alters insulin responses during glucose ingestion [32]. Specifically, statistically significant CpGs (FDR < 0.05) from our study that were previously related to insulin were located in the genes *LPL* [33], *GRB10* [34], *WISP1* [35], *PRDM16* [36], *TMEM132C* [37], *ADAMTS9* [38], and *NOX4* [39].

The CpG cg04615668 is located in the gene *CTNND2* (according to Illumina CG database), which encodes an adhesive junction associated protein called catenin delta 2, δ-catenin, NPRAP or neurojungin. This protein functions in Wnt signalling to regulate gene expression [40] and has been reported to be involved in the pathogenesis of cancer, cortical cataract-linked Alzheimer’s disease, autism, schizophrenia, mental retardation, myopia, and infectious diseases [40]. For example, *CTNND2* plays a critical role in neuronal development since it has been observed that it is likely rate-limiting for dendritic morphogenesis and maintenance, and its haploinsufficiency is common in autism [41]. However, little is known about the implication of *CTNND2* in metabolic diseases. In this context, a polymorphism located at this gene (rs6873671) has been significantly associated with human type 2 diabetes in two independent genome-wide studies [42,43], suggesting that *CTNND2* is involved in the regulation of glucose metabolism. Another polymorphism (rs10513097) has appeared in a genome-wide association study (GWAS) related to body mass index [44]. For this reason, it is necessary to highlight the importance of the present study, because it is the first time that methylation of this gene (in this case, DNA methylation in one CpG) has been linked with impairments in insulin sensitivity and glucose metabolism.

According to the current investigation, the association between cg04615668 methylation and CSi is negative, suggesting that hypomethylation of this site in PWBCs is related to higher insulin sensitivity. Moreover, methylation level in this CpG site is also correlated with two other insulin-related parameters such as the AIR index and HOMA-S, reflecting its involvement in insulin and glucose pathways.

On the other hand, the enzyme encoded by the *LPL* gene hydrolyses triglycerides in circulating chylomicrons, low density lipoproteins and very low density lipoproteins to render free unesterified fatty acids to the circulation [45]. *LPL* is synthesized in parenchymal cells such as skeletal muscle cells, adipocytes, macrophages and mammary gland cells, among other tissues and cell types [46]. After maturation in the rough endoplasmic reticulum (mainly driven by the lipase maturation factor-1 or LMF1), *LPL* is secreted and binds to heparan sulphate proteoglycans which are crucial in the translocation of the enzyme from its site of synthesis to the endothelium, also acting as cofactors in enzymatic reactions [47]. *LPL* activity is additionally regulated by apolipoproteins, angiopoietins, miRNAs and hormones. Insulin is considered a major regulator of adipose tissue *LPL*, through its effect on *LPL* transcription during adipocyte differentiation and through increasing *LPL* mRNA levels [48]. Initially, a tissue-specific regulation of *LPL* action by insulin was reported in such a way that *LPL* activity in adipose tissue was stimulated by acute infusions of insulin (leading to free fatty acids for storage) while *LPL* activity in skeletal muscle was decreased by this hormone [49]. However, nutritional studies involving 2 weeks of a high-carbohydrate diet or high-fat diet in human volunteers seemed to increase the *LPL* response to carbohydrate feeding in both adipose tissue and skeletal muscle [50]. Moreover, it is also important to remark that mice with muscle-specific *LPL* overexpression generated a muscle-selective insulin resistance [51]. In contrast, the disruption of *LPL* in skeletal muscle results in reductions in lipid storage and increased myocyte insulin signalling, together with marked insulin resistance in other tissues, leading finally to obesity and systemic insulin resistance. In support of a mediation role of *LPL* in systemic insulin sensitivity, Goodarzi et al. (2004) [52] and Goodarzi et al. (2007) [33] found that common *LPL* gene variation was involved in insulin resistance measured through hyperinsulinemic-euglycaemic clamps and intravenous glucose tolerance tests in Mexican Americans.

According to current research, the association between cg07263235 methylation and CSi is positive. Therefore, hypermethylation of this site in PWBCs might display higher insulin sensitivity. Methylation level in this CpG site is also positively correlated with the DI (CSi × AIR), showing that the hypomethylation in this site may indicate an impaired relative insulin secretion. Houde et al. described that *LPL* methylation in one specific CpG was lower in placentae of women with gestational diabetes mellitus [53]. However, there are other studies showing that an increase in *LPL* methylation was detrimental. Indeed, Castellano-Castillo et al. have described higher levels of *LPL* methylation in adipose tissue from patients with metabolic syndrome [54] and Drogan et al. showed an association between *LPL* methylation in adipose tissue and regional body fat distribution [55]. The disparity in the results from studies in the scientific literature is difficult to interpret given the multiple differential patterns of methylation in CpG sites of different cells and tissues.

Since the cg07263235 is located at the *LPL* promoter, a complementary analysis of putative transcription factors that bind on this CpG was performed using the software TRANSFAC (v2019.1) (GeneXplain, Wolfenbüttel, Germany). This software showed that cyclic AMP-responsive element-binding protein 1 (CREB1) may act in the regulation of *LPL* expression. Other investigators have demonstrated that glucose-dependent insulinotropic polypeptide (GIP), in the presence of insulin, upregulates adipocyte *LPL* gene transcription through CREB/cAMP-responsive CREB coactivator 2 (TORC2) activation [56]. Thus, we again speculate that the regulation of *LPL* by CREB might be mediated by cg07263235 methylation. Furthermore, it is worth noting that cg07263235 methylation also correlated with circulating creatinine in our study in a positive manner. Serum creatinine is a surrogate marker for muscle mass in healthy subjects [57]. Since skeletal muscle mass is inversely associated with T2D [58], low creatinine would represent a proxy of low muscle mass and possibly be linked to a higher risk of developing T2D [59]. 

Remarkably, both CpGs together allowed the distinction of individuals with low and high CSi with an AUC of 0.90. Since these CpGs are associated with different insulin-related parameters, it seems that although they are related to different glucose-related metabolic mechanisms, they complement each other to differentiate CSi groups. Therefore, we speculate on the hypothesis that the methylation at specific sites of the insulin-sensitive genes *CTNND2* and *LPL* may act as biomarkers of whole body insulin resistance, given a possible effect of DNA methylation on gene expression, with subsequent consequences in insulin resistance-related diseases. It must be noted that our population is very specific (healthy non-diabetic young women) and that the CSi is not usually measured in other investigations. Hence, these CpGs are more likely to be biomarkers of early diagnosis of possible insulin-related problems in a healthy population and not in diabetic or metabolic-impaired subjects. Indeed, after an exhaustive search of methylation databases and in a subpopulation of the Methyl Epigenome Network Association (MENA) study (*n* = 417, females = 59%, T2D = 59, non-T2D = 358), we have not been able to validate our CpGs in insulin-resistant individuals or with T2D (data not shown).

Our study presents several methodological limitations. The sample size is relatively small, which is partially a consequence of the complex IVGTT procedure. As it happens in association studies involving massive measurements, type I and type II errors cannot be discarded, although data preprocessing and strict CpG selections have been carried out to avoid them. Although methylation is tissue-specific, and methylation patterns and the study of insulin-sensitive organs, such as muscle or adipose tissue, are more appropriate to find epigenetic biomarkers for insulin sensitivity, the measure of DNA methylation biomarkers in white blood cells has the advantage of accessibility to the biological sample. Other studies have demonstrated that blood cells can act as proxies for these tissues [21,60,61]. Gene expression analysis would have been helpful to reveal the relationship between methylation and gene function in this particular study, but unfortunately, there was no biological sample available for this purpose. However, the association between the level of methylation in the CpG site cg04615668 and *CTNND2* gene expression is generally described as direct, whereas for cg07263235 methylation and *LPL* gene expression the relation was generally inverse, when assessed in the MEXPRESS online utility (https://mexpress.be/) based on multiple tissue gene expressions in several cancer types. Finally, causality cannot be established due to the transversal nature of the study. DNA methylation can either be a consequence, a cause, or a proxy of insulin action impairment.

In conclusion, this study reports for the first time an association between DNA methylation patterns with the insulin sensitivity index CSi measured through an intravenous glucose challenge. The most significant signals of association correspond to two CpGs located in the *CTNND2* (cg04615668) and *LPL* (cg07263235) genes. These findings may contribute to identifying potential biomarkers and new regulatory mechanisms in insulin-related diseases.

## 4. Materials and Methods

A cross-sectional study was carried out on 57 non-diabetic nulliparous, non-pregnant women volunteers without parental family history of diabetes. They were recruited for a metabolic study to assess future gestational diabetes (Table 1) (The GEDYMET Chilean study) [11]. Exclusion criteria were previous or in situ diagnosis of diabetes, family history of diabetes, dyslipidaemia, anaemia or pregnancy. The volunteers visited the Centre of Clinical Research (School of Medicine, Pontificia Universidad Católica de Chile) to carry out an abbreviated version of minimal-model IVGTT after the administration of 0.3 grams of glucose per kg of body weight, as a 50% water solution infused for 60 s [9]. As part of the abbreviated IVGTT protocol, plasma glucose and insulin levels were measured at −15, −5, 2, 3, 4, 5, 6, 8 and 10 min to calculate the AIR index as the area under the curve of plasma insulin [62]. After AIR, additional plasma glucose and insulin levels were measured at 10, 15, 20, 30, 40 and 50 min to complete IVGTT and to estimate the CSi using the website http://webmet.pd.cnr.it/csi/ [9]. CSi is considered a surrogate of insulin sensitivity showing strong association with the hyperinsulinemic-euglycaemic clamp. The IVGTT-based DI, represents a measure of insulin secretion adjusted by systemic insulin sensitivity and was calculated as the product of AIR × CSi [63]. Plasma glucose and insulin levels measured at −15 and −5 min before IVGTT were used to calculate the HOMA-S index, which is the inverse of the HOMA-IR index (HOMA-S = 1/HOMA-IR = 1/(fasting insulin (µUI/mL) × fasting glucose (mg/dL)/405)). This research was approved by the Ethics Committee of the School of Medicine, Pontificia Universidad Católica de Chile (Santiago, Chile) in compliance with the Helsinki Declaration of ethical principles for medical research involving human subjects (code 14-281, date 4th June 2015). All participants provided written informed consent.

### 4.1. Anthropometry, Blood Pressure and Biochemical Determinations

Anthropometric measurements were carried out by trained personnel in light clothing and without shoes, using a calibrated set of stadiometers, scales and tapes. Weight (kg) and height (m) were used to calculate BMI (kg/m^2^). Systolic and diastolic blood pressure (mmHg) were measured with digital sphygmomanometer as an average of three measurements. Venous blood samples were drawn by venipuncture in EDTA tubes. Plasma was separated from whole blood by centrifugation at 3500 rpm at 5 °C for 15 min, and frozen immediately at −80 °C until assay. Plasma levels of insulin (µU/mL) and glucose (mg/dL) were measured in the central laboratory of the Pontificia Universidad Católica de Chile by standard electro-chemiluminescence and colorimetric methods (http://redsalud.uc.cl/ucchristus/laboratorio-clinico/).

### 4.2. DNA Methylation Analysis

Genomic DNA was extracted from PWBCs using the MasterPureTM DNA purification kit (Epicenter, Madison, WI, USA) and quantified with the Pico Green dsDNA Quantitation Reagent (Invitrogen, Carlsbad, CA, USA). In order to convert cytosine into uracil, high-quality DNA samples (500 ng) were treated with sodium bisulfite using the EZ-96 DNA Methylation Kit (Zymo Research Corporation, Irvine, CA, USA) according to the manufacturer’s protocol. Illumina Infinium Human Methylation 450k BeadChip (Illumina, San Diego, CA, USA) was employed to measure DNA methylation levels of CpG sites across the human genome. This analysis was conducted in the Unidad de Genotipado y Diagnóstico Genético from the Fundación de Investigación Clínico de Valencia, as detailed elsewhere [64].

### 4.3. Treatment of Methylation Raw Data

Signal measurement intensities were scanned in the 450k array using the Illumina iScanSQ platform. The intensity of the images was extracted with the GenomeStudio Methylation Software Module (v 1.9.0, Illumina). Methylation raw data are available in NCBI’s gene expression omnibus [65] as part of the MENA study through GEO series accession number GSE115278 (https://www.ncbi.nlm.nih.gov/geo/query/acc.cgi?acc=GSE115278).

β-Values were computed using the formula β-Value = M/(U + M) where M and U are the raw “methylated” and “unmethylated” signals, respectively. β-Values were corrected for type I and type II bias using the peak-based correction. Data were normalized in R using a categorical subset quantile normalization method (SQN) and probes associated with X and Y chromosomes were filtered out using the pipeline developed by Touleimat and Tost [66]. Probes with single nucleotide polymorphisms (SNPs) were also filtered out. Differences in methylation resulting from differences in cellular heterogeneity were corrected using estimateCellCounts function from minfi package for R statistical software [67], based on the Houseman method [68].

### 4.4. Statistical Analysis

After pre-processing, in order to select CpGs with a higher effect that may present biological noticeable implications, 1416 CpGs were selected with a slope >|0.005| calculated from the relationship between methylation and CSi. The methylation of the 1416 CpGs was correlated with CSi using Spearman’s correlation coefficients. *p*-values were adjusted for multiple testing through the Benjamini–Hochberg method. Afterwards, the top 10 significant CpGs were selected, and the first two (cg04615668 and cg07263235) were further analysed. The Mann–Whitney U test was employed for evaluating the differences between two groups of individuals generated using the median of CSi. Logistic regressions and ROC curve AUCs, both adjusted by age, were calculated to determine if the CpGs were able to predict the median group of each individual. Correlations and the volcano plot were performed using the R statistical software [67]. Other statistical calculations, as well as the ROC curve, were performed with STATA version 12.0 (Stata Corp, College Station, TX, USA). The Manhattan plot, correlation graphs and box plots were generated using GraphPad Prism 6 (Graph-Pad Software, San Diego, CA, USA).

### 4.5. Ingenuity Pathway Analysis

After the selection of 1416 CpGs (see above), an adequate number of CpGs were selected by having Spearman correlations’ FDR < 0.05 (253 CpGs) and then, analysed using IPA software (Qiagen, Redwood City, CA, USA, www.ingenuity.com). Associated pathways and gene regulatory networks were identified by predefined pathways and functional categories of the ingenuity knowledge base [69]. Canonical pathway analyses were performed with IPA’s core analysis module and selected if *p* < 0.05 after Fisher’s test for multiple comparison was statistically significant.

## Figures and Tables

**Table 1:** Anthropometric and biochemical measurements, and insulin sensitivity indexes of *n* = 57 participants of this study.

Variable	N	Median (IQR)
Age (y)	57	25 (22–30)
Weight (kg)	57	59.5 (56.0–64.2)
Height (m)	57	1.59 (1.56–1.63)
Body mass index (kg/m^2^)	57	23.4 (21.8–25.4)
Plasma total cholesterol (mg/dL)	56	167.5 (148.0–196.5)
Plasma HDL cholesterol (mg/dL)	56	63.0 (51.5–71.5)
Plasma LDL cholesterol (mg/dL)	56	84.0 (67.5–103.5)
Plasma triglycerides (mg/dL)	56	92.5 (68.0–131.0)
Systolic blood pressure (mmHg)	57	113 (104–119)
Diastolic blood pressure (mmHg)	57	70 (65–75)
Fasting glucose (mg/dL)	49	78 (74–82)
Fasting insulin (IU/µmL)	49	7.0 (5.8–9.1)
HOMA-S	48	71.5 (55.5–88.5)
Calculated insulin sensitivity (CSi)	49	5.7 (4.0–8.2)
Acute insulin release (AIR)	49	538.7 (398.5–718.6)
IVGTT-based disposition index (DI)	49	2792.6 (2023.4–4136.4)

HDL: High density lipoprotein; HOMA-S: Homeostasis model assessment-insulin sensitivity; IQR: Interquartile range; IVGTT: Intravenous glucose tolerance test; LDL: Low density lipoprotein.

## Supplementary Materials

Supplementary materials can be found at https://www.mdpi.com/1422-0067/20/12/2928/s1.

## Abbreviations

AIR	Acute insulin response
AUC	Area under the curve
CREB1	Cyclic AMP-responsive element-binding protein 1
CSi	Calculated insulin sensitivity index
DI	Disposition index
EWAS	Epigenome-wide association study
FDR	False discovery rate
GEDYMET	Genetics, dysglycemia and metabolism
GIP	Glucose-dependent insulinotropic polypeptide
GWAS	Genome-wide association study
HOMA-IR	Homeostasis model assessment - insulin resistance index
HOMA-S	HOMA-insulin sensitivity index
IPA	Ingenuity pathway analysis
IVGTT	Intravenous glucose tolerance test
LPL	Lipoprotein lipase
MENA	Methyl Epigenome Network Association
PWBCs	Peripheral white blood cells
ROC	Receiver operating characteristic
SNP	Single nucleotide polymorphism
T2D	Type 2 diabetes
TORC2	CREB/cAMP-responsive CREB coactivator 2
