# Supplementary material for: Insulin Sensitivity Is Associated with Lipoprotein Lipase (LPL) and Catenin Delta 2 (CTNND2) DNA Methylation in Peripheral White Blood Cells in Non-Diabetic Young Women
Source: Int J Mol Sci. 2019 Jun 15;20(12):2928. doi: 10.3390/ijms20122928 (PMC6627674; doi:10.3390/ijms20122928)

**Supplementary Figure S1: Correlation of the methylation of the top 10 CpGs with the Calculated Sensitivity index (CSi).**


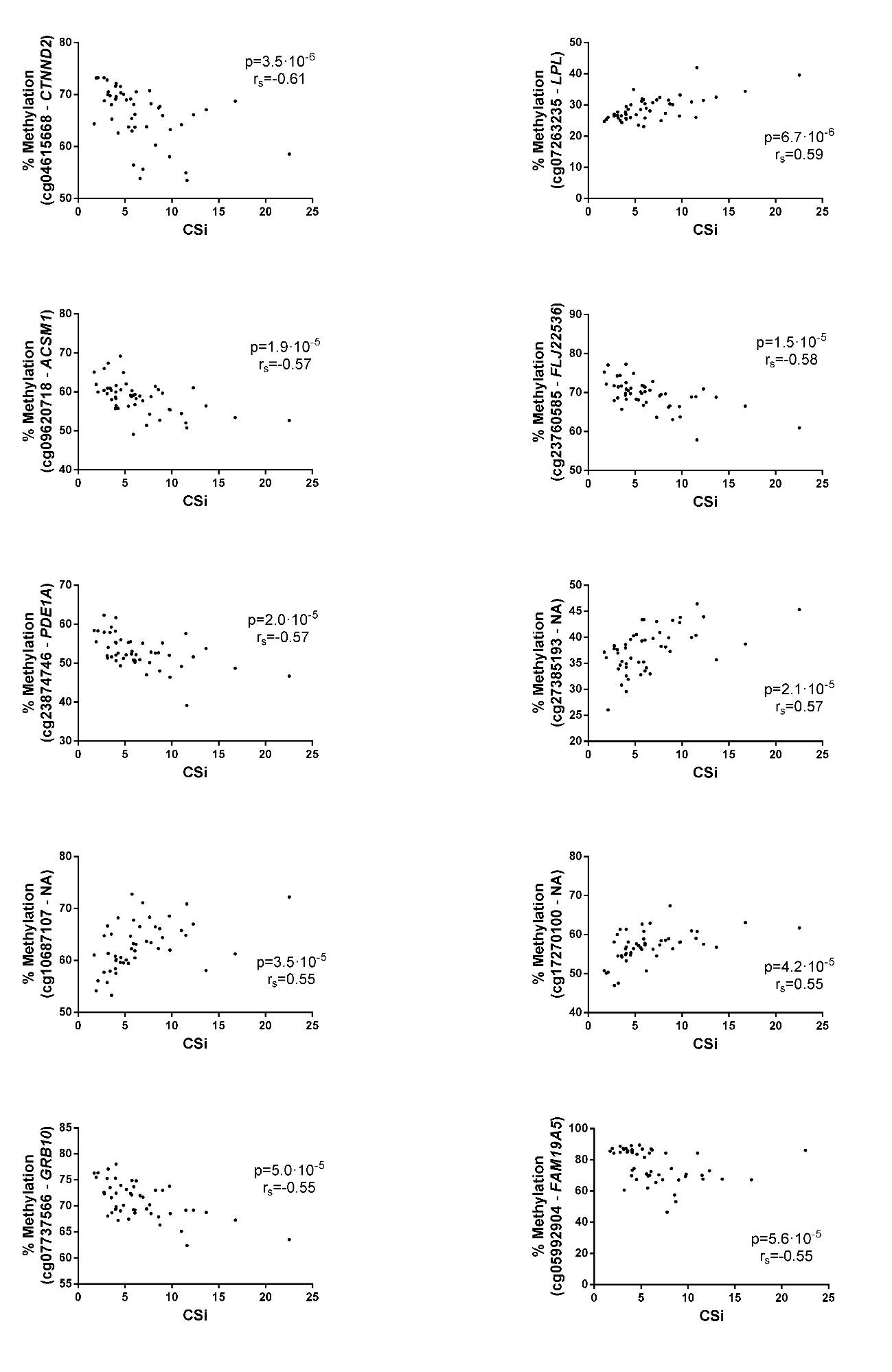

Supplement: Supplementary file 1 [file ijms-20-02928-s001.zip › Supplementary Figure S1.docx]
